# A Coordinate-Based Meta-Analysis of Overlaps in Regional Specialization and Functional Connectivity across Subjective Value and Default Mode Networks

**DOI:** 10.3389/fnins.2017.00001

**Published:** 2017-01-19

**Authors:** M. Yavuz Acikalin, Krzysztof J. Gorgolewski, Russell A. Poldrack

**Affiliations:** ^1^Graduate School of Business, Stanford UniversityStanford, CA, USA; ^2^Department of Psychology, Stanford UniversityStanford, CA, USA

**Keywords:** fMRI BOLD, metaanalysis, decision making, subjective value, default mode network

## Abstract

Previous research has provided qualitative evidence for overlap in a number of brain regions across the subjective value network (SVN) and the default mode network (DMN). In order to quantitatively assess this overlap, we conducted a series of coordinate-based meta-analyses (CBMA) of results from 466 functional magnetic resonance imaging experiments on task-negative or subjective value-related activations in the human brain. In these analyses, we first identified significant overlaps and dissociations across activation foci related to SVN and DMN. Second, we investigated whether these overlapping subregions also showed similar patterns of functional connectivity, suggesting a shared functional subnetwork. We find considerable overlap between SVN and DMN in subregions of central ventromedial prefrontal cortex (cVMPFC) and dorsal posterior cingulate cortex (dPCC). Further, our findings show that similar patterns of bidirectional functional connectivity between cVMPFC and dPCC are present in both networks. We discuss ways in which our understanding of how subjective value (SV) is computed and represented in the brain can be synthesized with what we know about the DMN, mind-wandering, and self-referential processing in light of our findings.

## 1. Introduction

Significant progress has been made in understanding the anatomy and function of the default mode network (DMN), which is characterized by robust task-induced decreases in activity in a consistent set of brain regions as well as robust correlations in activity during rest (Raichle and Snyder, 2007). Recently, the relation between the DMN and other previously identified functional networks for distinct cognitive processes has attracted increasing interest. This interest is in part driven by the fact that a variety of cognitive tasks reliably induce increased activity in DMN-associated regions, as opposed to the expected task-induced decreases in DMN activity. Cognitive processes that have been identified to elicit activity in DMN regions include emotional and social cognition, introspection, prospection, and autobiographical memory (Laird et al., 2009). These investigations have been valuable, as they provide insights into DMN function, which can help us understand what our brains are doing when we are “doing nothing” and how our brains deviate from this default state in response to task demands.

Here we focus on another set of cognitive processes that have been shown to entail task-driven increases of activity in a subset of DMN-related regions, namely value-based decision making. Specifically, a qualitative look at studies and reviews demonstrating the presence of a distinct network that plays a crucial role in the computation and representation of subjective value (SV) in value-based decision making (the subjective value network [SVN]) suggests overlaps with DMN in a number of regions core to both networks (Bartra et al., 2013; Clithero and Rangel, 2014). The goal of the present research is to quantitatively assess the nature and extent of overlap between the SVN and DMN. In doing so, we aim to synthesize our understanding of how SV is computed and represented in the brain with what we know about the DMN. We begin by shortly reviewing the separate literatures on each of these networks.

Understanding how the brain makes decisions and evaluates their outcomes is a core focus of neuroeconomics (Clithero and Rangel, 2014). A crucial step in the computational process of decision making is the assignment of value to the set of alternatives that is relevant to the decision at hand (Rangel et al., 2008). The need to make value-based decisions is shared across many animal species, as value-based decision making is necessary for simple animal behaviors like foraging as much as it is necessary for complex economic decisions made by humans in financial markets (Hayden et al., 2011).

There is consensus in the neuroeconomics literature around the presence of a distinct brain network responsible for the valuation process. The literature has provided strong evidence from both human and animal studies that the ventromedial prefrontal cortex (VMPFC) is involved in the assessment of SV (Rangel and Hare, 2010; Levy and Glimcher, 2012). The large corpus of publications and the variability of decision making contexts used in this line of research proves a challenge for the identification of a core brain network responsible for the computation of SV. Qualitative reviews of the literature (Rangel et al., 2008; Padoa-Schioppa, 2011) have proven useful, but a recent and highly informative trend has been conducting large coordinate-based meta-analyses (CBMA) in order to assess the full set of brain regions that correlate with the SV of potential actions across task and reward modalities (Bartra et al., 2013; Clithero and Rangel, 2014).

Two recent meta-analyses targeting the neuroeconomics literature with respect to the computation and representation of SV suggest that a core set of regions reliably co-activate and represent SV across task stages and outcome modalities, consisting of VMPFC, VSTR, and PCC (Bartra et al., 2013; Clithero and Rangel, 2014). This set of functionally connected regions, suggesting the presence of a distinct SVN in the brain, is further consistent with numerous qualitative reviews of the literature (Rangel et al., 2008; Glimcher, 2010; Grabenhorst and Rolls, 2011; Padoa-Schioppa, 2011).

Researchers outside neuroeconomics pursuing a variety of research programs in neuroimaging have observed reliable decreases in brain activity in a distinct set of brain regions during engagement in mental tasks (Raichle et al., 2001). These findings have attracted interest in the structure and function of this network, coined the DMN (Raichle and Snyder, 2007). Beyond task-related decreases, DMN has also been associated with increased activity during internally focused tasks involving self-referential thought, such as prospection, theory of mind, and autobiographical memory retrieval (Buckner et al., 2008). The withdrawal of activity from regions involved in the DMN, which include the VMPFC, PCC, precuneus (pC) inferior parietal lobule (IPL), lateral temporal cortex (LTF), dorsal medial prefrontal cortex (DMPFC), and medial temporal lobe (MTL) has been interpreted as the attenuation of internally focused, self-referential activity in the brain in order to better focus on the external demands of the environment and the task at hand (Sheline et al., 2009).

The relationship between DMN and other cognitive processes that lead to task-induced increases in DMN regions has recently attracted considerable interest. However, these investigations have mostly been focused on a specific group of cognitive processes most salient with our current conceptualization of the DMN. For example, Spreng et al. (2009) find common activation with DMN areas across autobiographical memory, prospection, and theory of mind tasks, concluding that the DMN supports the common denominator of these cognitive behaviors, which is the simulation of an internalized experience. The exact functional diversity and architecture of the DMN is far from understood. However, the regions involved in DMN are well defined and there is growing consensus that DMN plays a role in internally focused, self-referential processing that allows us to process our past and prepare for our future (Buckner and Vincent, 2007; Raichle and Snyder, 2007).

A noteworthy take on understanding the functional heterogeneity of the DMN using a meta-analytic approach is provided by Laird et al. (2009). Combining CBMA with behavioral domain and functional connectivity analyses, the authors build upon the insight that DMN may be responsible for not one, but multiple functions (Spreng et al., 2009). This insight is warranted given the task induced increases (as opposed to the expected decreases) in DMN regions during tasks used for studying processes, such as emotional and social cognition, decision making, introspection, prospection, and autobiographical memory (Laird et al., 2009).

One set of cognitive processes that drive activation in core DMN regions is related to value-based decision making (Bartra et al., 2013; Clithero and Rangel, 2014). However, the relation between the computation of SV in the brain (by the SVN) and the DMN has not been in empirical focus. Chavez et al. (2016) recently demonstrated that a classifier model built to distinguish positive and negative affective responses can also distinguish self-referential processing from thinking about others, most prominently in the VMPFC. This result is interesting to us as it links affective processes that are core to the SVN and value-based decision making to self-referential processing, which is widely considered as part of DMN function. In fact, two critical nodes of the DMN, namely VMPFC, and PCC, are also considered crucial in value-based decision making, as reviewed above. However, no publication to date has assessed the exact overlap in DMN and SVN-related activity across these regions. Understanding the overlap as well as dissociations between DMN and SVN within these regions can guide future research on how DMN and SVN are related, can inform our conceptualization of how SV is represented in the brain, and can improve our understanding of DMN function.

For these reasons, we conducted a series of CBMA's, addressing the questions about (1) whether and where there may be overlaps and dissociations across the SVN and DMN and (2) whether these overlapping regions also show similar patterns of functional connectivity within these networks. There are distinct advantages to using a CBMA-based approach in this investigation. A CBMA allows the definition of the exact extent of overlap between different domains, in addition to identifying subregions within regions of interests where these networks can be dissociated. In this case, it allows us to test whether there is complete overlap in VMPFC and PCC across DMN and SV-related activations, or whether there are distinct subregional specializations within VMPFC and PCC for both DMN and SVN. When combined with meta-analytic connectivity modeling, this approach can further shed light to functional connectivity between potentially overlapping subregions (in other words, overlapping functional subnetworks) across the two networks.

## 2. Materials and methods

We report the results from a series of CBMA, a widely used methodology in the neuroimaging literature (Eickhoff et al., 2009, 2012; Salimi-Khorshidi et al., 2009; Kober and Wager, 2010). We conducted CBMA using the activation likelihood estimation (ALE) method (Laird et al., 2005; Eickhoff et al., 2012; Fox and Friston, 2012; Turkeltaub et al., 2012). This approach is utilized in order to assess correspondence of neuroimaging results at the voxel level across a large number of studies (Laird et al., 2005). This methodology provides a test of the null hypothesis that activations reported in a set of studies are uniformly distributed across the brain, rather than concentrating in a subset of regions (Eickhoff et al., 2012).

Both the SVN and DMN have previously been subject to CBMA in separate contexts, aimed at identifying informatic parcellation within each network (Laird et al., 2009; Bartra et al., 2013; Clithero and Rangel, 2014). Qualitative and subjective observations of regional overlap between SVN and DMN have been noted in the recent literature (Clithero and Rangel, 2014), especially in VMPFC and PCC, regions that are associated with both networks. In this paper, we use the CBMA approach to provide a quantitative and objective assessment of whether there is such an overlap across these two networks and whether there are specialized subregions for each network within VMPFC and PCC. In addition to assessing overlaps in regional involvement across these two networks, we further assess overlapping patterns of functional connectivity using meta-analytic connectivity modeling (Laird et al., 2009; Robinson et al., 2010).

### 2.1. Selection criteria for CBMA

For the SVN dataset, we acquired (kindly provided by the authors) the results of a PubMed search for “fMRI” and “reward” conducted by Bartra et al. (2013), which was filtered based on a number of selection criteria. Sampling only English language papers that used BOLD fMRI, the authors excluded studies that use physical pain as a negative outcome, or those that use psychoactive drugs as a positive outcome. Further, they required that the study results reported a directional change in BOLD with respect to SV in the experimental task in stereotactic space (Talairach or MNI). The dataset excluded results based on region-of-interest (ROI) or small-volume-correction (SVC) analyses, such that the results under consideration involved the same threshold being applied uniformly across the whole brain. Finally, results included were only from healthy human participants (results in any given study corresponding to a patient population of interest were also excluded). See Bartra et al. (2013) for more details on selection criteria. This dataset provided by the authors included 348 studies from 206 publications. This corpus of publications used a wide variety of experimental tasks with varying elements of choice, risk, learning, and numerous different outcomes, including rewards and penalties in primary, monetary, social, and other contexts (Bartra et al., 2013). We converted the subset of results reported in Talairach space to MNI space using the Lancaster (icbm2tal) transformation (Lancaster et al., 2007).

For the DMN dataset, we conducted a BrainMap search using the same search criteria used in Laird et al. (2009) to access published task independent activations, using Sleuth 2.3. The search criteria were (1) “Normal Mapping” context (healthy participants only), (2) “Low-Level Control” experimental controls (resting or fixation conditions only), and (3) “Deactivations” (contrasts in which the signal during baseline was greater than during task). The matching results, all extracted in MNI stereotactic coordinates, included 181 studies from 89 publications. Thus, this dataset of contrasts involving regions more active at “rest” compared to “task” spanned a wide variety of paradigms.

We queried and transformed the provided spreadsheet of SV data from Bartra et al. (2013) into GingerALE input format using a custom Python script (accessible at https://git.io/vPNed). We included activation foci for only positive SV effects, omitting contrasts involving deactivations in response to increasing SV. Further, we excluded activation foci from the “wait” stage activations (activity between decision stage and outcome receipt stage), leaving only activations during decision and outcome. We included all outcome types (monetary, primary, and others). This resulted in an ALE input including 2942 foci from 285 experiments. For the BrainMap search results for DMN data, we used 1577 foci from all 181 experiments.

### 2.2. ALE meta-analysis

A widely used method in the literature, the ALE approach to conducting CBMA's involves testing against the null hypothesis that activation foci in the dataset are distributed uniformly across the brain, thus looking for regions with above chance concentration of activity across studies (Laird et al., 2005; Eickhoff et al., 2012). In other words, the ALE approach provides for each voxel in the brain an activation likelihood estimate, which is the probability that at least one activation focus from the set of experiments truly lies at that voxel based on Gaussian principles of spatial uncertainty (Bartra et al., 2013).

The computation of ALE statistics involves three main steps. First, the probability of activation in any given voxel is computed as a function of three variables: the Euclidean distance between the reported peak coordinates and the voxel, the volume of the voxel (8mm^3^ here), and the spatial uncertainty associated with the reported peak coordinates. These are mapped onto a modeled activation map (MA). Second, MA maps are aggregated across contrasts by taking the probabilistic union of the MA statistic across all contrasts for each voxel, returning the ALE statistic. Finally, a permutation test is used to identify voxels where the ALE statistic is higher than that expected by chance (Laird et al., 2005; Eickhoff et al., 2012; Clithero and Rangel, 2014). We performed the ALE analyses in MNI space, using GingerALE 2.3.5 for the CBMA and GingerALE 2.3.6 for connectivity modeling.

We started by computing two ALE maps for DMN and SVN data sets separately, thresholding the ALE map with a voxel-level requirement of *p* < 0.001 and a cluster-level requirement of *p* < 0.05. The significance levels of ALE values were determined by comparing the resulting ALE statistics to a null distribution generated from 10, 000 permutations as outlined above.

Second, we carried out contrasts between the results from the individual analyses of SVN and DMN datasets in order to determine if there were any differences in the spatial pattern of loci across the two ALE maps. This analysis resulted in a conjunction map indicating regions of overlap, as well as two contrast maps indicating dissociable regions where either SVN or DMN was more correlated with the reported loci. Differences between ALE maps were also compared to a null distribution generated by 10, 000 permutations in order to generate *p*-values, and we implemented a false discovery rate (FDR) correction at the voxel-level for the *p*-values at the *p* < 0.05 level.

After determining the location of regional overlaps between the DMN and SVN using the approach explained above, we used meta-analytic connectivity modeling (MACM) in order to investigate whether thee functional connectivity patterns of these overlapping regions also overlap across the two networks. By assessing groups of coordinates that co-activate across a large number of experiments, CBMA's can be used to identify functionally connected networks in the brain. This is done by identifying the specific ROI, seeding these regions of interest back to the original data sets in order to identify all other studies and their foci that also reported activation within the ROI, and computing ALE statistics for only this subset of foci. Put simply, the meta-analytic approach and the statistical procedure remains the same, but applied only on a subset of the corpus of studies that report activation within the ROI. Based on assessing the spatial co-occurrence of spatially separate neurophysiological events, this approach can be used to identify functional connectivity (Rogers et al., 2007; Laird et al., 2009). This approach has been used previously to assess the functional connectivity of the amygdala (Robinson et al., 2010), and informatic parcellation in both the DMN (Laird et al., 2009) and the SVN (Clithero and Rangel, 2014).

## 3. Results

All results reported here can be accessed as unthresholded ALE maps online (accessible at http://neurovault.org/collections/1653/). The results for single-domain analyses for SVN and DMN were consistent with previous analyses in the original papers by Bartra et al. (2013) and Laird et al. (2009), as well as the findings of a number of other meta-analyses on both research domains in the literature (Schilbach et al., 2008; Clithero and Rangel, 2014). Moreover, the comparisons made between SVN and DMN-related activity by the contrasts and conjunction between the two ALE maps demonstrated overlaps and dissociations within regions involved in both DMN and SVN. Finally, the meta-analytic connectivity analyses found evidence for functional connectivity between PCC and VMPFC in both networks. Specifically, PCC activity was associated with wide regional co-activations within VMPFC, while VMPFC activity alone was associated with relatively limited volumes of PCC co-activation, indicating some asymmetry in the functional connectivity between VMPFC and PCC.

### 3.1. Subjective value

The CBMA found five clusters of convergence significantly correlated with increasing SV across all included studies. These regions included the striatum, bilateral amygdala, VMPFC, dorsal and ventral PCC, in addition to the superior frontal gyrus (SFG). The cluster containing the maximum ALE statistic was in a large cluster containing striatum, VMPFC, and dACC (ALE = 251.5 × 10^−3^). The results are presented in Table S1 and Figure S1.

The only region where our results, as well as Bartra et al. (2013)'s results, diverge from Clithero and Rangel (2014)'s findings on the SVN is the activity found in the left SFG; but it is worth noting that this cluster disappears with the use of only slightly more stringent thresholding, suggesting that it is not as robust as the other results reported here.

### 3.2. Default mode network

The CBMA of DMN foci suggested a large number of clusters, including the pC, PCC, DMPFC, VMPFC, bilateral IPL, bilateral MTG, left middle frontal gyrus (MFG), bilateral hippocampus, claustrum, and striatum. The cluster containing the maximum ALE statistic was in the VMPFC (ALE = 79.8 × 10^−3^). The results are presented in Table S2 and Figure S2.

Unlike Laird et al. (2009), we do find convergence in the hippocampus in DMN, which may reflect the availability of more than a dozen new studies relevant to our search criteria in the BrainMap database compared to 2009. This suggests that the increased power of the analysis with added studies may have resolved some issues concerning susceptibility artifacts or high spatial variability associated with task related deactivations in this area.

One exception to the convergence between our results and Laird et al. (2009) is the presence of small clusters in the striatum, which could have arisen from the less stringent thresholding used here. However, the main purpose of the present investigation is not the single-domain analyses, but an analysis of regions related to both SVN and DMN; thus, the application of less conservative thresholding at this stage was warranted by our purpose of assessing overlaps between the two networks. The second round of thresholding during the contrast analyses helps increase the stringency of results to compensate for these less stringent thresholds at the first stage.

### 3.3. SVN-DMN conjunction and contrasts

**Figure 1:**
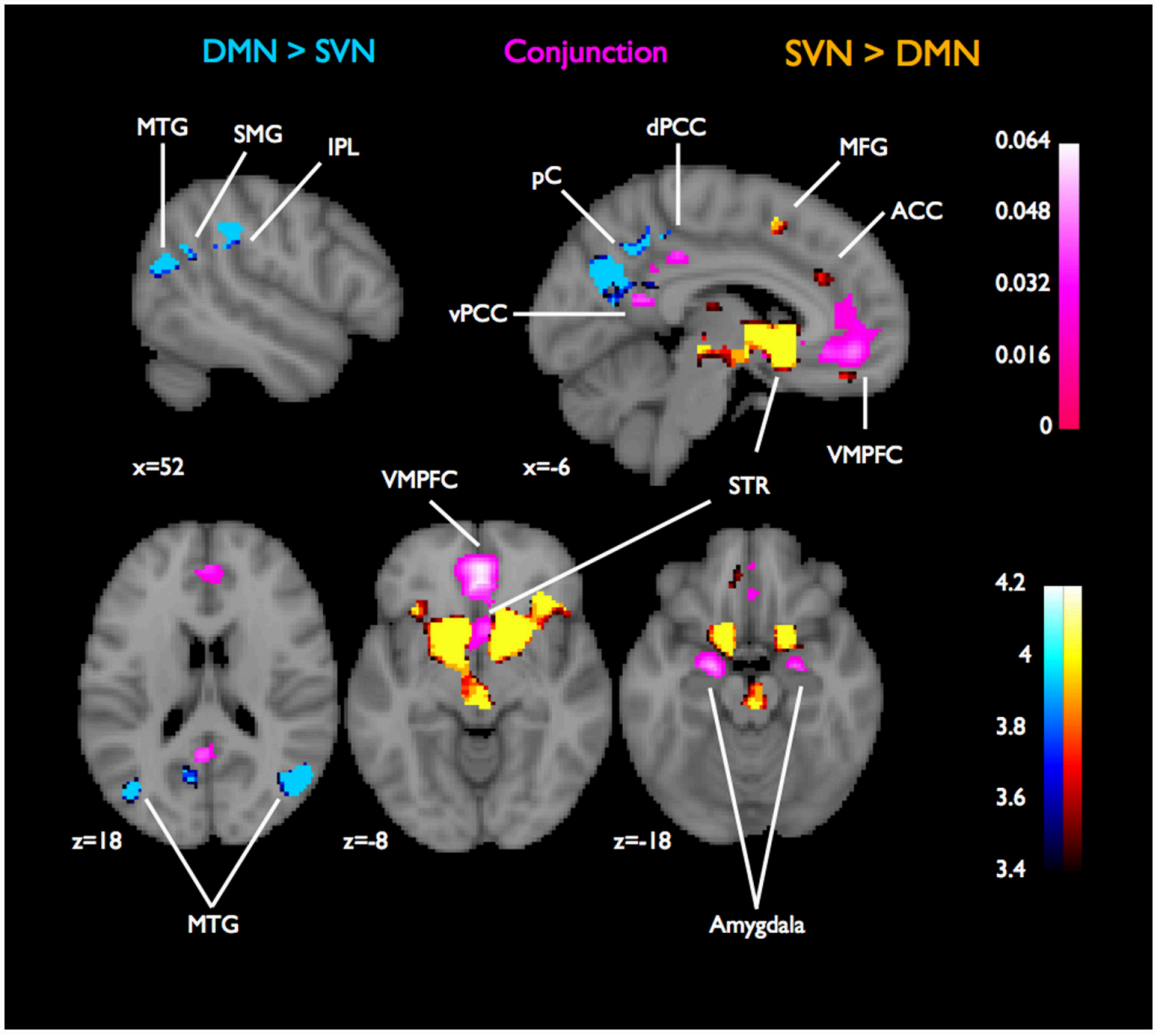
**Conjunctions and contrasts of DMN and SVN using CBMA**. For assessing functional specialization for DMN or SVN, we contrasted their ALE images, which are reported as z-scores (in blue for DMN specialization or red for SVN specialization). The overlaps were assessed by the conjunction of the DMN and SVN ALE images and are reported as ALE scores (in purple). Overlaps across the two networks were found in VMPFC, striatum, ventral and dorsal PCC, and bilateral amygdala. The overlap cluster containing the maximum ALE statistic was in the VMPFC (ALE = 64.4 × 10^−3^). Coordinates and cluster information for the conjunction are listed in Table 1.

The conjunction analysis of SVN and DMN ALE maps revealed eight clusters in regions of overlapping activity across these two networks. These clusters were in VMPFC, striatum, ventral and dorsal PCC, and bilateral amygdala. The overlap cluster containing the maximum ALE statistic was in the VMPFC (ALE = 64.4 × 10^−3^). These results are presented in Table 1 and Figure 1.

**Table 1:** **Maxima and cluster information for the conjunction of CBMA results for SVN and DMN**.

**Cluster**	**Volume (*mm*^3^)**	***x***	***y***	***z***	**ALE (× 10^−3^)**	**Region**
		***(weighted center)***				
1	11344	0.7	42.2	−2.3	64.39	VMPFC & Anterior Cingulate
2	1800	1.6	11.3	−7.1	43.15	Striatum
3	1312	−1.4	−38.2	36.1	46.38	Posterior Cingulate
4	1152	−20.2	−5.5	−17.5	52.35	Left Amygdala
5	616	−2.4	−52.6	16.9	44.88	Posterior Cingulate
6	440	24.9	−5.6	−17.6	42.89	Right Amygdala
7	24	−8	24	−4	25.89	Striatum
8	16	−5	22	−4	24.62	Striatum

*The analysis identified eight distinct clusters of overlap using ALE. Results are shown in Figure 1*.

The two especially surprising results are the overlaps found in striatum and amygdala. Both of these regions, despite being closely involved in valuation-related processes (Hare et al., 2008; Seymour and Dolan, 2008), are not considered core parts of the DMN.

Furthermore, these results demonstrate that two main areas of interest that are widely considered parts of both the SVN and the DMN, namely VMPFC and PCC, diverge in the amount of overlap observed across these two domains. A large proportion of MPFC, clustered around central ventromedial prefrontal cortex (cVMPFC), shows non-specialization across the SVN and DMN, with only a small part of aVMPFC activating selectively in the SV dataset. In dorsal posterior cingulate cortex (dPCC), there is also considerable overlap between SVN and DMN, while a number of clusters in the more ventral areas of PCC activate selectively in the DMN contrasts. These results indicate that while activity in especially more central regions of the VMPFC is hardly dissociable across these two networks, there seems to be some regional specialization for DMN in PCC. Meanwhile, the areas of PCC that the SVN recruits almost completely overlap with DMN-associated activity in the PCC.

There were multiple clusters in the LMTG, RMTG, PCC, and pC where the likelihood of activation (DMN-SVN contrast) was greater for DMN than SVN (See Figure 1). On the other hand, activity in one large cluster centered in the striatum extending to thalamus and hypothalamus, as well as some small clusters in insula, anterior VMPFC (aVMPFC), and SFG were more correlated with SVN than DMN (SVN-DMN contrast, see Figure 1).

### 3.4. Meta-analytic connectivity modeling

Of specific interest, supported by the results of the first step of our analysis, was the regional overlap between the default mode and SVN around VMPFC and PCC. For this reason, for each network, we investigated the set of areas that exhibit reliable co-activation with VMPFC and PCC. We hypothesized that VMPFC and PCC might demonstrate functional connectivity with each other in both networks. To test this hypothesis, we constructed two spherical 12 mm ROIs near the cVMPFC and dPCC, based on regions exhibiting local maxima in the results of the earlier analyses. The VMPFC ROI was centered at (*x,y,z*) = (−2, 44, −4) and the dPCC ROI was centered at (*x,y,z*) = (−2, −36, 38). Then, we subsetted the corpus of studies to only include studies reporting activation foci within these ROIs. The VMPFC ROI identified 487 foci from 31 experiments in the DMN dataset, and 772 foci from 69 experiments in the SV dataset. The dPCC ROI identified 424 foci from 27 experiments in the DMN dataset, and 450 foci from 36 experiments in the SV dataset.

**Figure 2:**
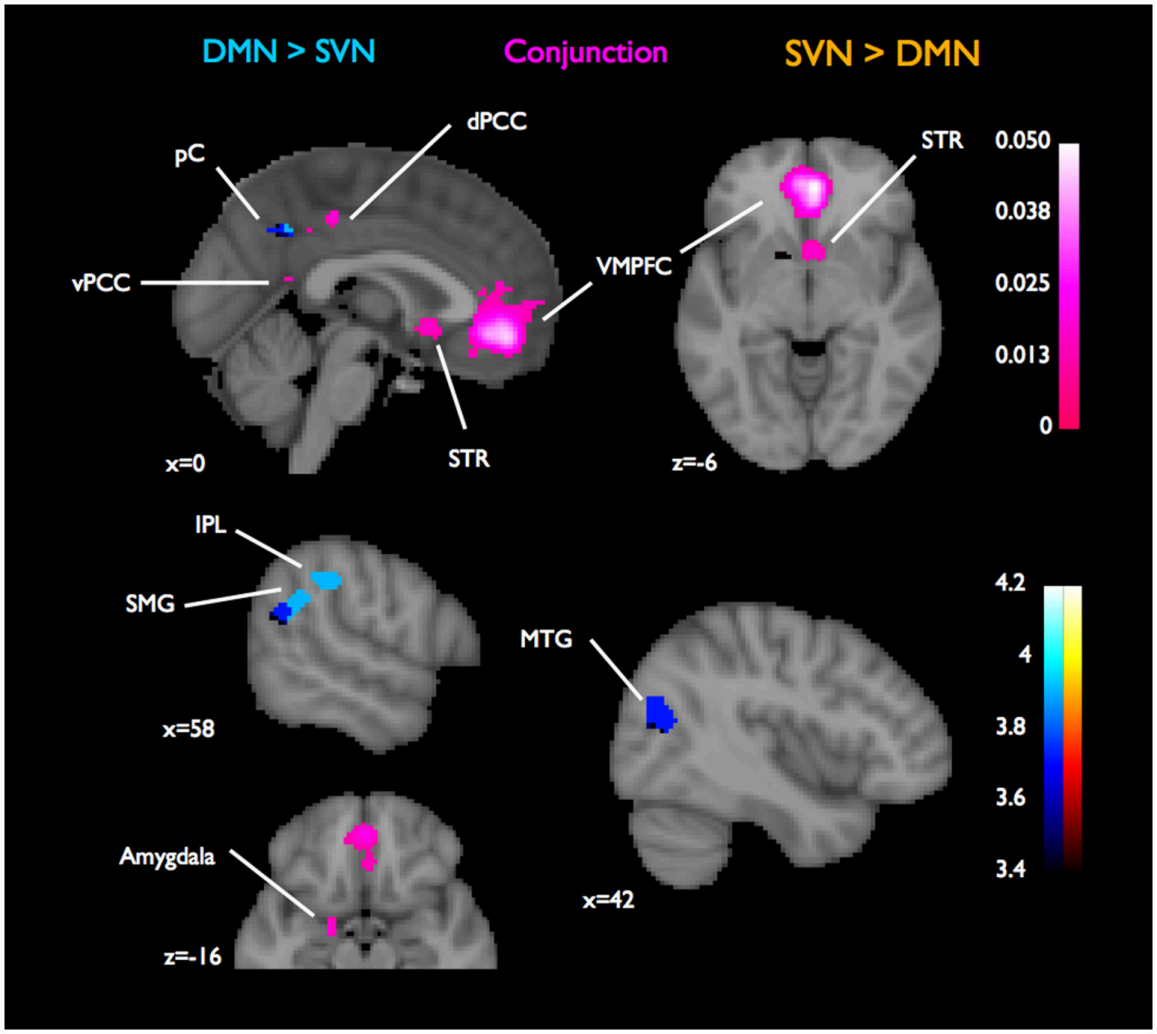
**Conjunctions and contrasts of DMN and SVN using MACM, focusing on functional connectivity with VMPFC**. For assessing connectivity patterns that were specific to DMN or SVN, we contrasted their ALE images, which are reported as z-scores (in blue for DMN-specific connectivity or red for SVN-specific connectivity). The overlaps in functional connectivity assessed by the conjunction of the DMN and SVN ALE images and are reported as ALE scores (in purple). Overlaps in functional connectivity with VMPFC were found in dPCC, vPCC, striatum, amygdala, and MFG. The maximum ALE statistic outside the VMPFC seed in this conjunction was found in dPCC (ALE = 20.1 × 10^−3^). Coordinates and cluster information for the conjunction are listed in Table 2.

The areas that showed co-activation with VMPFC in DMN were primarily dPCC, vPCC, pC, and the amygdala. In addition, other clusters of co-activation were found in bilateral MTG, RIPL, LMFG, striatum, and right supramarginal gyrus (RSMG). The cluster containing the maximum ALE statistic outside the VMPFC was in the dPCC (ALE = 31.4 × 10^−3^). The results are presented in Table S3 and Figure S3. On the other hand, in SVN, co-activations with VMPFC were found in the striatum, dPCC, vPCC, LMFG, right insula, and thalamus. The cluster containing the maximum ALE statistic outside the VMPFC was in the striatum (ALE = 103.3 × 10^−3^). The results are presented in Table S4 and Figure S4. A conjunction analysis of these two co-activation maps revealed 9 clusters of overlap, including dPCC, vPCC, striatum, bilateral MFG, and left amgydala. The maximum ALE statistic outside the VMPFC in this conjunction was found in dPCC (ALE = 20.1 × 10^−3^). The results are presented in Table 2 and Figure 2.

**Table 2:** **Maxima and cluster information for the conjunction of MACM results for SVN and DMN looking at functional connectivity with VMPFC**.

**Cluster**	**Volume (*mm*^3^)**	***x***	***y***	***z***	**ALE (× 10^−3^)**	**Region**
		***(weighted center)***				
1	10736	0.1	43.2	−5.5	50.12	VMPFC & Anterior Cingulate
2	720	2.4	11.1	−5.4	18.46	Striatum
3	232	0.8	−32.1	43.3	20.06	Posterior Cingulate
4	200	−4.1	−52.2	16.4	19.93	Posterior Cingulate
5	184	−16.9	−2.3	−16	19.37	Left Amygdala
6	112	3.3	−40.6	38.3	14.87	Posterior Cingulate
7	32	4	57	11	16.44	Right Medial Frontal Gyrus
8	16	2	57	8	14.06	Left Medial Frontal Gyrus
9	8	2	−50	18	12.79	Posterior Cingulate

*The analysis identified nine distinct clusters of overlap using ALE, eight outside of the VMPFC seed. Results are shown in Figure 2*.

Looking at the contrasts between the two ALE maps of connectivity with VMPFC, six clusters in grey matter passed the second threshold for showing distinct connectivity with VMPFC in the DMN. These clusters were located in the precuneus, bilateral MTG, RIPL, RSMG, and LMFG. The cluster containing the maximum ALE statistic was in the RIPL (ALE = 354.0 × 10^−3^). For SVN, the only cluster indicating distinct functional connectivity with VMPFC was found in the striatum (ALE = 389.1 × 10^−3^). See Figure 2.

**Figure 3:**
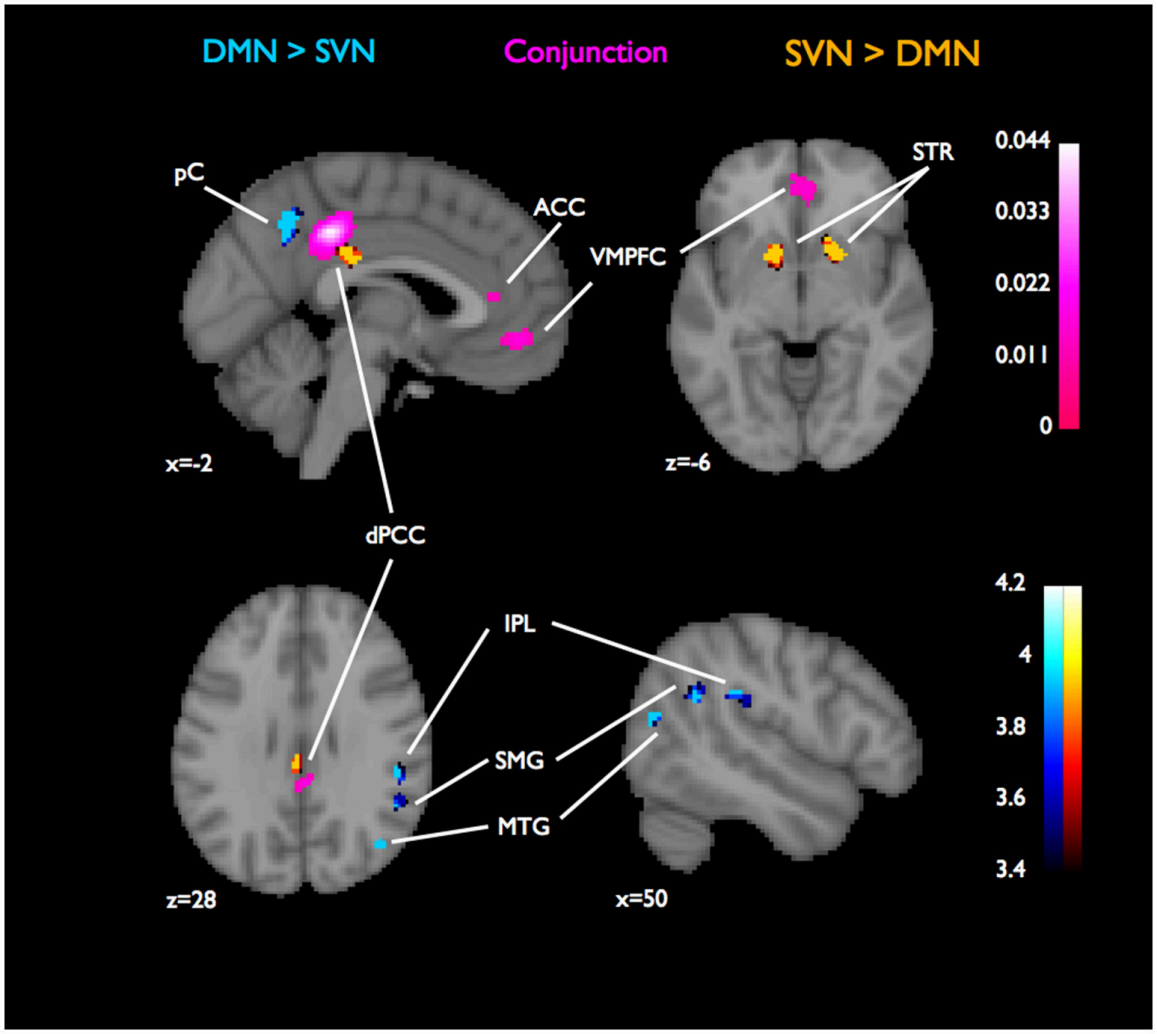
**Conjunctions and contrasts of DMN and SVN using MACM, focusing on functional connectivity with dPCC**. For assessing connectivity patterns that were specific to DMN or SVN, we contrasted their ALE images, which are reported as z-scores (in blue for DMN-specific connectivity or red for SVN-specific connectivity). The overlaps in functional connectivity assessed by the conjunction of the DMN and SVN ALE images and are reported as ALE scores (in purple). Overlaps in functional connectivity with PCC were found in VMPFC and dACC. The maximum ALE statistic outside the dPCC seed in this conjunction was found in the dACC (ALE = 20.4 × 10^−3^). Coordinates and cluster information for the conjunction are listed in Table 3.

We then looked at co-activations with dPCC in both networks. In DMN, clusters of activity in the VMPFC, pC, dACC, LMFG, RIPL, and bilateral MTG were found to co-activate with dPCC. The cluster containing the maximum ALE statistic outside the dPCC was in RMTG (ALE = 31.1 × 10^−3^). The results are presented in Table S5 and Figure S5. In the SV data, we found clusters of co-activations with dPCC in the striatum, VMPFC, dACC, right inferior frontal gyrus, right insula, and midbrain. The cluster containing the maximum ALE statistic outside the dPCC was in the striatum (ALE = 50.0 × 10^−3^). The results are presented in Table S6 and Figure S6. Looking at the conjunction of these two co-activation maps, we found clusters in the VMPFC and dACC indicating overlap in functional connectivity between dPCC, dACC, and VMPFC across the two networks. The maximum ALE statistic outside the dPCC in this conjunction was found in dACC (ALE = 20.4 × 10^−3^). The results are presented in Table 3 and Figure 3.

**Table 3:** **Maxima and cluster information for the conjunction of MACM results for SVN and DMN looking at functional connectivity with PCC**.

**Cluster**	**Volume (*mm*^3^)**	***x***	***y***	***z***	**ALE (× 10^−3^)**	**Region**
		***(weighted center)***				
1	4472	−1.1	−36.4	38.2	43.73	Posterior Cingulate
2	1424	0.4	44.6	−6.5	18.43	VMPFC
3	496	2.5	41.8	11	20.37	Anterior Cingulate
4	56	11.2	45.4	6.6	10.48	VMPFC
5	8	8	46	4	12.21	Anterior Cingulate

*The analysis identified five distinct clusters of overlap using ALE, four outside of the PCC seed. Results are shown in Figure 3*.

The contrast between the PCC-connectivity ALE maps for the two networks revealed that DMN had distinct co-activations between PCC, pC, RIPL, and RMTG. See Figure 3. The cluster containing the maximum ALE statistic was in the pC (ALE = 389.1 × 10^−3^). On the other hand, in the SVN, PCC was found to have distinct co-activations in the striatum and a small subregion of PCC (ALE = 389.1 × 10^−3^). See Figure 3.

## 4. Discussion

Our goal in this research was to investigate whether and where there may be regional overlaps and dissociations across the SVN and DMN and to assess whether these overlapping regions also show similar patterns of functional connectivity. We found considerable overlaps between SVN and DMN in cVMPFC and dPCC. Moreover, our findings show that similar patterns of functional connectivity between dPCC and cVMPFC are present in both networks. In SVN as well as DMN, activation in dPCC alone seems to elicit co-activation in a large and overlapping subregion of cVMPFC. On the other hand, activity in cVMPFC alone leads to a smaller set of subregions showing co-activation in the dPCC. Thus, there is some asymmetry in the bidirectional functional connectivity between cVMPFC and dPCC, such that a large portion of cVMPFC seems to be an important component of computations that involve dPCC in both DMN and SVN, while a relatively limited set of subregions in dPCC engage as consistently along with computations that involve cVMPFC across the two networks.

This pattern of regional overlap in these two crucial regions of interest is consistent with the more central (as opposed to anterior or ventral) subregion of VMPFC that Clithero and Rangel (2014) found to consistently co-activate with dPCC and VSTR. This suggests that of the two patterns of co-activation with central VMPFC that were previously identified in the SVN [the other being co-activations in vPCC and left angular gyrus (LAG)], the cVMPFC-dPCC co-activations seem to be present in DMN as well, while VMPFC-vPCC-LAG co-activations are not. This finding is also consistent with recent work dissociating the functions of dPCC and vPCC, demonstrating increasing connectivity within the DMN for dPCC and decreasing connectivity within the DMN for vPCC with increasing task difficulty (Leech et al., 2011). The overlaps we find in dPCC across SVN and DMN thus provide further evidence that dPCC may serve as an interface between the DMN and other networks that modulate externally directed behavior (Vincent et al., 2008; Laird et al., 2009; Margulies et al., 2009; Leech et al., 2011; Utevsky et al., 2014).

While reporting their results, Clithero and Rangel (2014) point out the similarity of SVN with subnetworks found within the DMN, and speculate that value computations that require higher levels of episodic memory and mental simulations may be likely to recruit regions associated with DMN, such as dPCC. The results here suggest that there might be some validity to this hypothesis, especially given Clithero and Rangel (2014)'s finding that activation in PCC is more strongly correlated with decision values than outcome values. This finding is informative, because if there is a demand for mental simulations in the process of value-based decision making, it must be more prominent during the decision stage, when outcomes are still uncertain and a state of the world resulting from a decision has not yet been realized. Further in support of this idea, Bartra et al. (2013) find strong clustering of activity in PCC for monetary, but not primary outcomes. In fact, PCC has received relatively little attention in the neuroeconomics literature with respect to SV. The results of the meta-analysis here suggest that SV-related activations in the dPCC overlap with those related to the DMN. dPCC may be involved in integrating mental simulations generated by the DMN aimed at assessing more abstract potential outcomes during the valuation process. In many decision contexts (decision-making under risk and uncertainty, intertemporal choice, anticipations of reciprocated social rewards in altruistic behavior) the potential outcomes are uncertain or intangible and thus might require internally focused and complex simulations of future states of the world for their valuation.

An alternative point of view could be raised based on findings relating PCC activation to task engagement, change detection, and monitoring of choice-irrelevant value. Pearson et al. (2011) propose a unifying perspective reconciling numerous mental activities that PCC has been associated with, including guiding memory and movement (Vogt et al., 1992), reward outcome monitoring (McCoy et al., 2003), goal-directed cognition (Spreng et al., 2010), action evaluation and behavioral modification (Hayden et al., 2008), and late stages of reinforcement learning (Bussey et al., 1996). This pattern of functional claims is consistent with PCC being engaged in change detection, suggesting that this might be a core function of the DMN (Pearson et al., 2011). In a similar vein, Grueschow et al. (2015) show that PCC tracks choice-independent value while VMPFC tracks choice-dependent value, and further show that the strength of PCC activation predicts attentional capture by choice-irrelevant stimuli. These findings suggest that the PCC (and its default-mode associated functions) might play an important role in value-based decision making, especially in terms of behavioral control and the evaluation of affordances (Pearson et al., 2011; Grueschow et al., 2015). Our finding of a shared cVMPFC-dPCC subnetwork between DMN and SVN can not differentiate between these two accounts. However, there may not be such a need, as these two accounts of the role PCC might play in the SVN (assessing abstract outcomes or assessing alternative courses of actions) and preexisting explanations for the role of PCC in the DMN have a common denominator, which is the integration of mental simulations into other cognitive processes. In these terms, this idea is also consistent with Leech et al. (2012), where the authors provide evidence for dPCC's role as a cortical hub integrating information from different functional networks in the brain. In fact, Buckner et al. (2008) propose that within the DMN, the MTL provides information from previous associations and memories, which serve as the building blocks of mental simulation, while MPFC to facilitates the use of these building blocks in the construction of self-referential mental simulations. According to this perspective, these subsystems converge in the PCC, where information is integrated.

It is also important to consider the anatomical overlaps in VMPFC activation foci across DMN and SVN, and whether they may point toward overlaps in the function of VMPFC across these networks. In addition to being a component of both SVN and DMN, VMPFC has been shown to play a role in affect regulation by interpreting affective information and altering responses based on contextual demands (Schiller and Delgado, 2010; Giustino and Maren, 2015). VMPFC has also been associated with social cognition, and is theorized to play a role in social judgments by simulating mental states that evaluate one's own and others' behaviors (Hughes and Beer, 2012; Flagan and Beer, 2013; Schurz et al., 2014). It is appealing to unify these functions into a more basic and fundamental role. Interestingly, there are overlaps in DMN and the roles that are attributed to VMPFC within these distinct perspectives. As we discussed before, the tracking of internal states and the integration of memory and prospection for simulating abstract rewards are important from the SV perspective. The affective regulation perspective has provided evidence that VMPFC may modulate the interpretation and responses to affective stimuli by redefining their meaning through the integration of value and contextual information (Delgado et al., 2016). This perspective is associated with self-referential cognition and also presents a potential need for the integration of information from processes responsible for memory, prospection, and planning that are associated with the DMN. Finally, the social cognition perspective argues for VMPFC involvement in the simulation of mental states for evaluating self- and other-generated social behavior, which follows the same theme. For these reasons, in line with Buckner et al. (2008), the cVMPFC-dPCC subnetwork we have found to be overlapping across DMN and SVN may warrant characterization as potentially being responsible for generating information based on mental simulations in cVMPFC and integrating them in dPCC into various more specialized processes that guide internally- or externally-directed behavior, such as economic or social decision-making. Although the overlaps between social cognition and DMN lie beyond the scope of this paper, future research could assess similarities and differences between DMN, valuation, and social cognition.

It is worthwhile acknowledging the local parcellation of activity in VMPFC for different cognitive functions. For example, Clithero and Rangel (2014) present some evidence for a posterior-to-anterior activation gradient in VMPFC as one moves from concrete to abstract reward modalities, in line with Grabenhorst and Rolls (2011). On the other hand, the results of Bartra et al. (2013) suggest a unified neural system that represents SV across different outcome modalities. Given the ostensible similarities in their datasets, these divergent results may be driven by small differences their exclusion criteria (exclusive focus on studies that report parametric effects of SV or including those reporting only high vs. low value) or their utilization of slightly different meta-analytic tools (Activation Likelihood Estimate and Multilevel Kernel Density Analysis). Yet, both analyses also find a cluster in more anterior VMPFC that co-activates with vPCC, which we find to be specialized for SVN. Therefore, it would be inaccurate to classify the entirety of VMPFC as simply and only a subcomponent of DMN. In a similar vein, the SV, social cognition, and affective regulation related functions of VMPFC can be partially dissociated into distinct subregions within the VMPFC (Zhang et al., 2015; Delgado et al., 2016).

It is worth thinking about this pattern of overlap from the DMN perspective as well, especially given some overlap we also found in the striatum across the two networks. Past work has shown that SV signals can be found in the brain even when consumer products are placed outside the focus of attention (Tusche et al., 2010), in contexts where SV of products are irrelevant to the choice at hand (Grueschow et al., 2015), or when there is no choice involved at all (Smith et al., 2014; Lebreton et al., 2015). The involvement of a common cVMPFC-dPCC circuit in DMN and SVN could alternatively be indicative of an inherent and automatic tendency of the brain to track value representations. Keeping track of SV in simulations of the past and the future might be important in learning from the past and better preparing for the future.

Our analyses also suggested that activity in LMTG, RMTG, and pC is significantly more correlated with DMN, while activity in the aVMPFC and striatum is significantly more correlated with SVN. In fact, this aVMPFC cluster is almost identical to the latter VMPFC subnetwork identified by Clithero and Rangel (2014) that co-activated with the left angular gyrus and vPCC instead of with dPCC. Activity in this anterior part of the VMPFC, in our analyses as well as in Bartra et al. (2013) and Clithero and Rangel (2014) is most closely related with the processing of monetary rewards, and shows little activity during both decisions and outcomes with primary rewards. This suggests that there might be functional specialization in aVMPFC for valuation. Bartra et al. (2013) suggest that aVMPFC could be involved in the mapping the value of abstract outcomes, such as monetary and social rewards (which, based on our results, may be relayed by the dPCC-cVMPFC subnetwork) to the same common currency space for comparisons across outcome modalities. Furthermore, we found parcellation within the PCC with a large proportion of it corresponding to DMN function, consistent with the heterogeneity previously found in this region (Leech et al., 2011, 2012; Utevsky et al., 2014).

In conclusion, our meta-analysis provides direct quantitative evidence demonstrating regional overlaps between the SVN and DMN in the brain. In addition, our findings point toward a shared functional cVMPFC-dPCC subnetwork. The possibility of this shared subnetwork serving the same function across the two networks raises a wide range of thought-provoking questions about what the common functional denominator of DMN and SVN might be. Our results, based on our understanding of the literature, suggests that the cVMPFC-dPCC subnetwork may be present in various cognitive processes that require the generation and integration of mental simulations, involving autobiographical memory, prospection, and self-referential thought. Future research could investigate the specific role of self-referential processes and mental simulations of the past and the future in the assessment and representation of abstract rewards. A specific question of interest is how different subregions of PCC may change their patterns of connectivity with the rest of the brain as rewards become more and more abstract during value-based decision making. Based on our results, we would speculate that one would observe increased connectivity of dPCC with the rest of the SVN as rewards become more abstract. Furthermore, future research could look at whether automatic SV representations play a role in spontaneous mind wandering at rest. Finally, it is an open question whether this cVMPFC-dPCC subnetwork may be active in other cognitive processes, potentially integrating information from mental simulations for more optimal behavior. Such cognitive processes could include social behaviors, such as cooperation (Fehr and Fischbacher, 2004) and moral judgment (Greene and Haidt, 2002) or planning for goals (Van der Linden et al., 2003).

## Author contributions

MYA acquired and assembled input data, conducted analyses, generated reports, and prepared the manuscript. KG and RP. supervised the project at all stages and edited the manuscript.

### Conflict of interest statement

The authors declare that the research was conducted in the absence of any commercial or financial relationships that could be construed as a potential conflict of interest.
