# Supplementary material for: A Coordinate-Based Meta-Analysis of Overlaps in Regional Specialization and Functional Connectivity across Subjective Value and Default Mode Networks
Source: Front Neurosci. 2017 Jan 19;11:1. doi: 10.3389/fnins.2017.00001 (PMC5243799; doi:10.3389/fnins.2017.00001)
Supplement: Supplementary file 1 [file DataSheet1.pdf]

Supplementary Material: A Coordinate-Based Meta-Analysis of Overlaps in Regional Specialization and Functional Connectivity Across Subjective Value and Default Mode Networks

Mehmet Yavuz Acikalin [1,*], Krzysztof Jacek Gorgolewski [2] and Russell Alan Poldrack [2]

*Correspondence:
Mehmet Yavuz Acikalin
655 Knight Way
Stanford, CA 94041
USA
mya@stanford.edu

1 SUPPLEMENTARY TABLES AND FIGURES

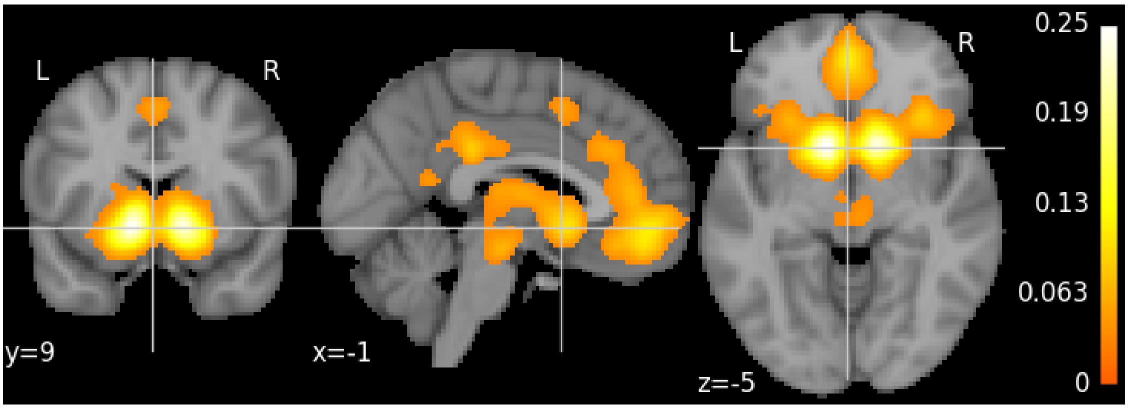

Figure S1. CMBA results for SVN data. Results are reported as ALE scores. Coordinates and cluster information for the conjunction are listed in Table S2.

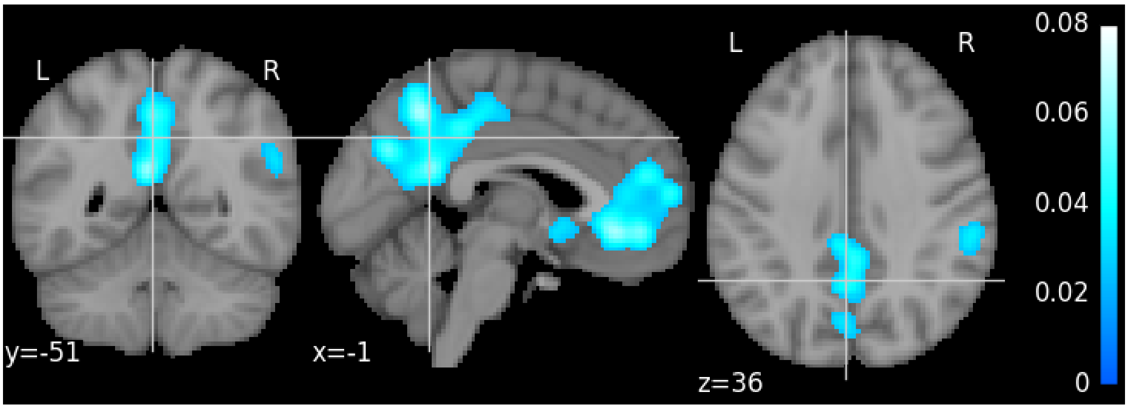

Figure S2. CMBA results for DMN data. Results are reported as ALE scores. Coordinates and cluster information for the conjunction are listed in Table S1.

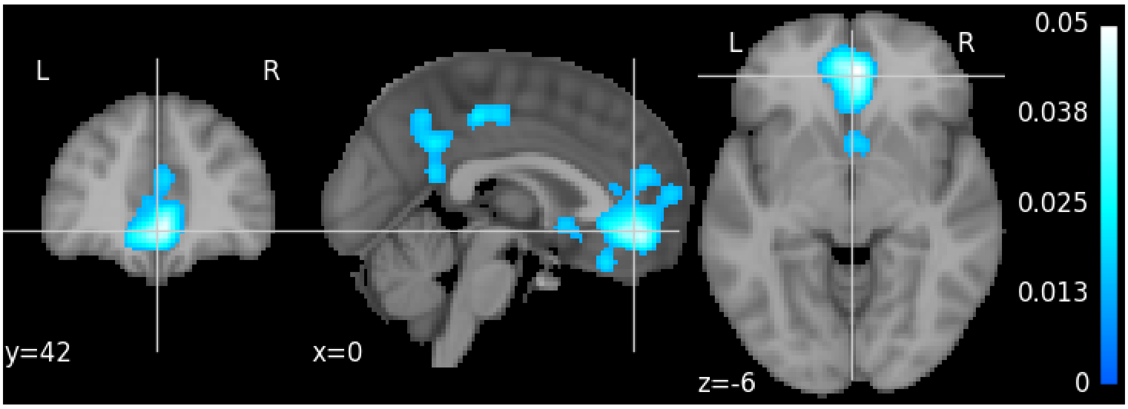

Figure S3. MACM results for DMN data looking at functional connectivity with cVMPFC. Results are reported as ALE scores. Coordinates and cluster information for the conjunction are listed in Table S3.

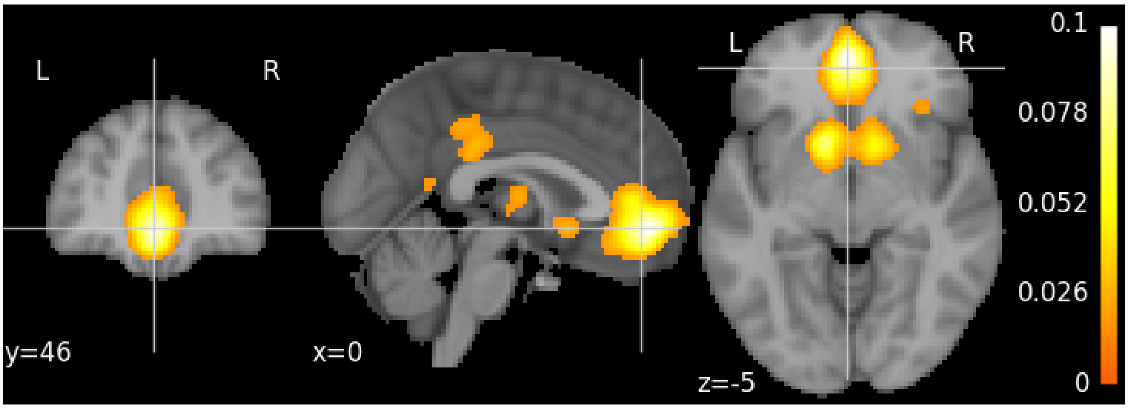

Figure S4. MACM results for SVN data looking at functional connectivity with cVMPFC. Results are reported as ALE scores. Coordinates and cluster information for the conjunction are listed in Table S4.

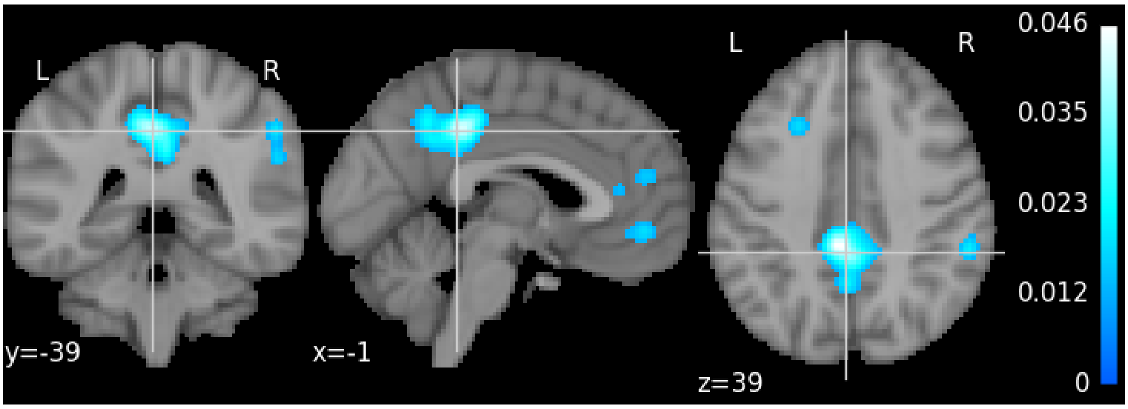

Figure S5. MACM results for DMN data looking at functional connectivity with dPCC. Results are reported as ALE scores. Coordinates and cluster information for the conjunction are listed in Table S5.

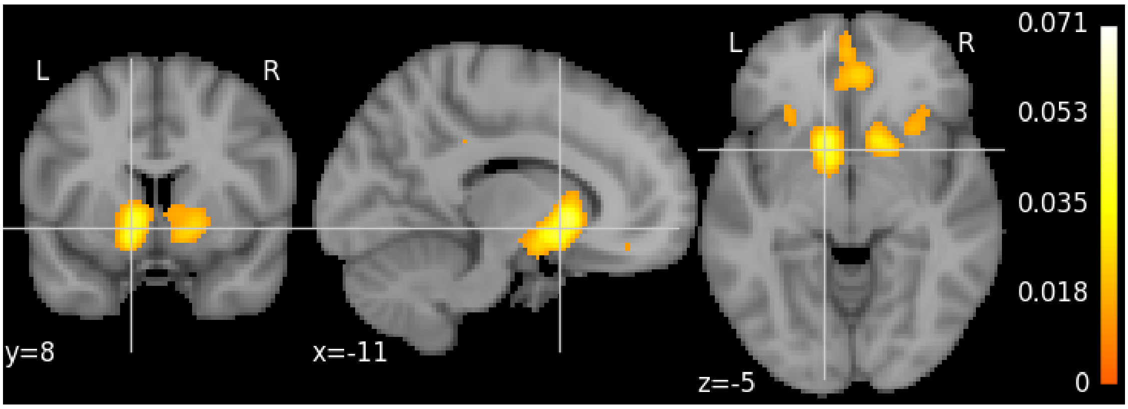

Figure S6. MACM results for SVN data looking at functional connectivity with dPCC. Results are reported as ALE scores. Coordinates and cluster information for the conjunction are listed in Table S6.

Cluster	Volume (mm^3)	x	y	z	ALE ($\times 10^{-}3$)	Region
1	91504	0.1	15.3	-3.5	251.52	Striatum & VMPFC & Anterior Cingulate
2	4144	-0.7	-30.6	32.6	85.73	Posterior Cingulate
3	1704	-0.8	10.2	49	53.27	Superior Frontal Gyrus
4	1416	-22.2	29.3	48.7	58.59	Left Superior Frontal Gyrus
5	1104	0.3	-51.9	16.1	47.22	Precuneus

Table S1. Maxima and cluster information for the CBMA results for SVN data. The analysis identified five distinct clusters of overlap using ALE. Results are shown in Figure S2.

Cluster	Volume (mm^3)	x	y	z	ALE ($\times 10^-3$)	Region
1	26008	-0.9	-51.4	35.8	65.82	Precuneus & Posterior Cingulate
2	17760	0.3	42.3	0.3	79.78	VMPFC & Anterior Cingulate
3	6456	47.5	-62.2	20.2	57.74	Right Middle Temporal Gyrus
4	4072	-42.8	-70.8	22	47.66	Left Middle Temporal Gyrus
5	2656	-22	-7.3	-18.9	52.97	Left Hippocampus
6	2472	55.3	-31.1	31.7	38.83	Right Inferior Parietal Lobule
7	2112	-26.2	15.8	44.4	48.99	Left Middle Frontal Gyrus
8	1984	1.7	11.8	-7.6	43.15	Striatum
9	1632	25.6	-9.8	-19.5	46.92	Right Hippocampus
10	984	-57.8	-34.8	28.3	38.01	Left Inferior Parietal Lobule
11	856	43.5	-7.5	-13.4	34.4	Claustrum
12	712	29.6	26.6	44.6	30.84	Right Middle Frontal Gyrus
13	640	43.6	-10.4	5.6	29.27	Right Insula
14	536	-21.7	48.8	7.7	32.84	Right Middle Frontal Gyrus

Table S2. Maxima and cluster information for the CBMA results for DMN data. The analysis identified fourteen distinct clusters of overlap using ALE. Results are shown in Figure S1.

Cluster	Volume (mm^3)	x	y	z	ALE ($\times 10^-3$)	Region
1	15408	0.3	43.4	-2.8	50.12	VMPFC & Anterior Cingulate
2	4736	-0.6	-51.6	32.1	31.37	Posterior Cingulate & Precuneus
3	2584	-22.8	-5.4	-18.4	30.90	Left Amygdala
4	1976	52.8	-31.5	27.9	24.19	Right Inferior Parietal Lobule
5	1792	43.5	-69	18.4	30.62	Right Middle Temporal Gyrus
6	1496	1.3	-24.5	46.1	23.28	Posterior Cingulate
7	1192	22.2	-7	-17.6	24.70	Right Amygdala
8	1120	-40.5	-71.6	18.6	25.46	Left Middle Temporal Gyrus
9	1080	-24.9	15.8	44.6	26.58	Left Middle Frontal Gyrus
10	1040	56.5	-50	24.4	19.32	Right Supramarginal Gyrus
11	904	-44.6	-2.7	-18.3	19.21	Left Middle Temporal Gyrus
12	856	2.4	12.1	-6.3	18.46	Striatum

Table S3. Maxima and cluster information for MACM results for DMN looking at functional connectivity with cVMPFC. The analysis identified twelve distinct clusters of overlap using ALE, eleven outside of the cVMPFC seed. Results are shown in Figure S3.

Cluster	Volume (mm^3)	x	y	z	ALE ($\times 10^-3$)	Region
1	30808	-1.6	32.7	-5.2	103.34	VMPFC & Anterior Cingulate & Striatum
2	2144	0	-33.1	35.5	29.99	Posterior Cingulate
3	1320	-19.5	32.2	49.3	29.86	Left Superior-Middle Frontal Gyrus
4	1112	-0.4	-52.6	14.8	27.79	Posterior Cingulate
5	1096	3	-12.1	7.1	26.93	Thalamus
6	872	34.1	23.2	-10.3	24.47	Right Insula

Table S4. Maxima and cluster information for MACM results for SVN looking at functional connectivity with cVMPFC. The analysis identified six distinct clusters of overlap using ALE, five outside of the cVMPFC seed. Results are shown in Figure S4.

Cluster	Volume (mm^3)	x	y	z	ALE ($\times 10^-3$)	Region
1	10576	-0.9	-40.3	39	46.45	Posterior Cingulate & Precuneus
2	3704	54.4	-41.8	29.1	24.13	Right Inferior Parietal Lobule
3	2264	1.3	45	-4.8	18.78	VMPFC
4	2184	0.6	43.4	14	22.85	Anterior Cingulate
5	1672	43.8	-68.9	17.9	31.05	Right Middle Temporal Gyrus
6	1096	-41.3	-69.9	19.2	20.97	Left Middle Temporal Gyrus
7	864	-23.9	18.2	44.2	25.07	Left Middle Frontal Gyrus
8	856	-19.6	49.8	8.7	17.09	Left Medial Frontal Gyrus

Table S5. Maxima and cluster information for MACM results for DMN looking at functional connectivity with dPCC. The analysis identified eight distinct clusters of overlap using ALE, seven outside of the dPCC seed. Results are shown in Figure S5.

Cluster	Volume (mm^3)	x	y	z	ALE ($\times 10^-3$)	Region
1	7888	-12.7	8.5	-5.3	50.00	Striatum
2	7056	-0.9	-33.3	35.1	70.52	Posterior Cingulate
3	6696	1.7	42.3	-2.8	29.18	VMPFC & Anterior Cingulate
4	3272	13.8	10.9	-4.5	35.02	Striatum & Right Insula
5	2360	31.7	21.1	-12.4	25.93	Right Inferior Frontal Gyrus
6	856	2.1	-20	-16.2	22.90	Midbrain

Table S6. Maxima and cluster information for MACM results for SVN looking at functional connectivity with dPCC. The analysis identified six distinct clusters of overlap using ALE, five outside of the dPCC seed. Results are shown in Figure S6.
